# Point-of-Care Ultrasound—History, Current and Evolving Clinical Concepts in Emergency Medicine

**DOI:** 10.3390/medicina59122179

**Published:** 2023-12-15

**Authors:** Joseph Osterwalder, Effie Polyzogopoulou, Beatrice Hoffmann

**Affiliations:** 1 Polipraxis, 9000 St. Gallen, Switzerland; 2 Emergency Medicine Department, Attikon University Hospital, 12462 Athens, Greece; effiepol@med.uoa.gr; 3 Department of Emergency Medicine BIDMC, One Deaconess Rd., WCC2, Boston, MA 02215, USA

**Keywords:** ultrasound, POCUS, emergency medicine, diagnosis, clinical sonography

## Abstract

Point-of-care ultrasound (PoCUS) has become an indispensable standard in emergency medicine. Emergency medicine ultrasound (EMUS) is the application of bedside PoCUS by the attending emergency physician to assist in the diagnosis and management of many time-sensitive health emergencies. In many ways, using PoCUS is not only the mere application of technology, but also a fusion of already existing examiner skills and technology in the context of a patient encounter. EMUS practice can be defined using distinct anatomy-based applications. The type of applications and their complexity usually depend on local needs and resources, and practice patterns can vary significantly among regions, countries, or even continents. A different approach suggests defining EMUS in categories such as resuscitative, diagnostic, procedural guidance, symptom- or sign-based, and therapeutic. Because EMUS is practiced in a constantly evolving emergency medical setting where no two patient encounters are identical, the concept of EMUS should also be practiced in a fluid, constantly adapting manner driven by the physician treating the patient. Many recent advances in ultrasound technology have received little or no attention from the EMUS community, and several important technical advances and research findings have not been translated into routine clinical practice. The authors believe that four main areas have great potential for the future growth and development of EMUS and are worth integrating: 1. In recent years, many articles have been published on novel ultrasound applications. Only a small percentage has found its way into routine use. We will discuss two important examples: trauma ultrasound that goes beyond e-FAST and EMUS lung ultrasound for suspected pulmonary embolism. 2. The more ultrasound equipment becomes financially affordable; the more ultrasound should be incorporated into the physical examination. This merging and possibly even replacement of aspects of the classical physical exam by technology will likely outperform the isolated use of stethoscope, percussion, and auscultation. 3. The knowledge of pathophysiological processes in acute illness and ultrasound findings should be merged in clinical practice. The translation of this knowledge into practical concepts will allow us to better manage many presentations, such as hypotension or the dyspnea of unclear etiology. 4. Technical innovations such as elastography; CEUS; highly sensitive color Doppler such as M-flow, vector flow, or other novel technology; artificial intelligence; cloud-based POCUS functions; and augmented reality devices such as smart glasses should become standard in emergencies over time.

## 1. Introduction

### 1.1. “You Have to Know the Past to Understand the Present”—Carl Sagan

Traditional diagnostic ultrasound was developed in the 20th century [1]. After some crucial technological advances in the 1960s and 1970s, two different approaches to diagnostic ultrasound began to emerge [2]. Some medical systems began using ultrasound (US) imaging services that were usually embedded in the specialty of radiology, with few other specialties developing a significant and consultative ultrasound practice. Over time, and with increasing ultrasound use in those particular regions, radiologists and even cardiologists started to employ technicians to obtain images, especially once it became a real-time imaging tool and required learning hands-on skills. However, this approach came to the detriment of large numbers of physicians being able to develop adequate hands-on skills in ultrasound, and it likely caused this type of practice model to function as a consultative imaging test. These exams were seldom performed by the treating physician and hence had to use very standardized approaches for imaging certain areas of the body. The physician requesting the test was rarely present during the exam. Hence, opportunities to ad hoc modify or tailor exams in real time or change or broaden the clinical questions of an exam were very limited. Obviously, exams that would require serial imaging and in patients with very fluid and dynamic clinical presentations (one of the best aspects of real-time ultrasound) are not feasible with this approach.

In parallel to these developments, other countries created a very different approach to diagnostic ultrasound, which was likely due to profound differences in medical practice and health care delivery. In this setting, the treating physician was encouraged to perform their own ultrasounds, and the concept of the “technician” was not embraced. In the mid-20th century, this parallel approach was the domain of internists, gynecologists, surgeons, but also cardiologists, and radiologists [3,4,5,6]. Diagnostic ultrasounds were performed by the treating physician skilled in hands-on image acquisition. This included the clinical interpretation of the findings. Over time, many other specialists joined the ranks of ultrasound-practicing physicians in countries where this differing concept was practiced [7,8,9,10]. Naturally, right from the beginning, ultrasound examinations were often already embedded in the clinical examination, so the performing physician was able to adapt and modify the exam ad hoc. One could make the argument that this was the earliest phase of “clinical ultrasound” and to some degree already included the concept of “point-of-care” sonography. Needless to say, more and more physicians recognized ultrasound’s diagnostic capabilities and began to practice physician-performed ultrasound. For instance, by the 1990s, diagnostic ultrasound was so common in some countries using this practice model that many primary care physicians, internists, or obstetrics/gynecologists had sonography equipment in their private offices and incorporated ultrasound scans as part of their patients’ clinical visits.

Physician-performed diagnostic ultrasound became so common in some health care systems that occasionally, efforts were undertaken to evaluate pathways to avoid the overuse of diagnostic ultrasound [11]. As a remarkable contrast, systems that mainly practiced technician-based ultrasound and limited ultrasound hands-on practice to much fewer specialties seemed to experience a relative decrease in overall diagnostic ultrasound performance. Compared to computed tomography or MRI, diagnostic ultrasound started to lose its share of imaging studies. For instance, a study by Liebeskind et al. based in a large tertiary care radiology department evaluated the causes for a decline in diagnostic ultrasound referrals by primary care physicians. The lack of knowledge of the diagnostic capacity of ultrasound among referring physicians was identified as the major factor [12]. This worldwide dichotomy of diagnostic ultrasound practice patterns is fascinating and still present. It is, in our view, an intriguing curiosity about medical practice in the 20th and early 21st centuries.

However, driven by technological advancements in the early 1980s, a third practice model for diagnostic ultrasound emerged. Surgeons, and eventually emergency medicine physicians of the new specialty of emergency medicine, began to use focused sonography in acute trauma patients in the shock room [13,14]. This was possible because of newer equipment allowing for more mobile and smaller machines with expanded processor capacity. These machines could be positioned next to the patient, moved by physicians to different exam spaces, and allowed for dedicated real-time imaging in acutely ill patients at their bedside. In the shock room, this method was initially limited to the rapid detection of trauma-related hemoperitoneum [15]. However, true portable and hand-held ultrasound machines were already utilized by 1978 and were employed by specialists outside the shock room in the 1980s [16]. Focused point-of-care exams were included in these evaluations. For instance, a two-dimensional echocardiography study by Schwarz and Meltzer in 1988 reported an impressive 96% accuracy in diagnosing cardiac wall motion abnormalities in patients hospitalized for acute ischemic cardiac events who experienced recurring chest pain episodes during admission. This study was conducted with a handheld portable device that weighed 3.3 pounds, had a 2 h battery life, and eventually had the capacity to print images. Bedside cardiology, obstetrics, and even residual bladder volume evaluations at the point of care were performed [17].

During those early years, the newly formed specialty of emergency medicine (EM) quickly recognized the diagnostic potential of ad hoc bedside and portable sonography [18], and one could argue that the need to define new methods of physician-performed ultrasound at the point of care was greatest in countries practicing a technician-based ultrasound model. Ultrasound, with its ability to answer pressing clinical questions in real time, especially in unstable or undifferentiated patients with acute complaints, made it the perfect diagnostic candidate in these settings. Initially, use was focused on identifying causes of hemodynamic instability in medical and trauma patients, procedure guidance, or patients presenting with undifferentiated abdominal, back, and chest pain [19,20]. Gradually, EM physicians expanded their use of sonographic point of care. Soon, the term emergency medicine ultrasound (EMUS) was used to describe exams that were exclusively performed by physicians, similar to the above-described physician-based ultrasound concept but using a laser-focused diagnostic approach. Emergency physicians (EPs) in both types of practice models (technician-performed vs. physician-performed ultrasound) started to include EMUS in their scope of practice. Not surprisingly, EPs began introducing the EMUS approach into systems where US was traditionally physician-performed, and physicians of other specialties who already practiced or understood the “point of care” approach (and likely already tailored their own exams to clinical presentations and point of care) had very different implementation experiences and hurdles to overcome compared to EPs practicing in technician-dependent systems with a paucity of specialists performing their own exams and unfamiliarity with this ultrasound concept [21].

Ultrasound is a technology that not only captures real time images, but is also able to visualize the real-time status of the living person, and the “life” of the patient [22]. One of the most profound examples is likely parents (as well as the sonographer) being able to see an unborn child’s movements and heartbeat [22]. However, it is also a technique that requires two human beings to interact in very close proximity. Naturally, this interaction includes many layers of additional interpersonal communication allowing the sonographer physician to capture a more holistic view of the patient, to talk to their body, and to understand the uniqueness of the person using their intuition, improvisation, and their capability to react to individuality [22]. Therefore, it makes great sense that the attending physician performs the examination themselves. This approach has the advantage that all the intangible layers of information will not be lost. In our opinion, the ultrasound-guided dialogue with the living body is a simple, but also the best way, to penetrate into the depths of the hidden pathologies of our patients, which are revealed ever so gradually over the course of the examination.

In the present, PoCUS and EMUS, as we practice it, is an established bedside diagnostic or interventional type of sonography performed and interpreted by adequately trained and directly for patient-caring physicians. It is documented with stored images and reports, just like any other diagnostic imaging modality, and its comprehensiveness and completeness are adapted to the clinical question and the individual patient. A large number of clinical studies, systematic reviews, and position and consensus papers form the basis for the many recommendations by experts and professional societies that the entire range of PoCUS can, should, and must be used in emergency patients, and that underline its usefulness. The following examples are a selection of the state of the art of EMUS documented with relevant citations out of the abundant studies available. The list of indications for EMUS has increased significantly in recent years, and its benefits for improving the clinical evaluation of patients and the performance of interventions have been proven many times over [23,24,25,26,27,28,29,30,31,32,33,34,35]. There is also a lot of evidence that EMUS speeds up consultation processes [36,37,38]. PoCUS is also often used as part of clinical pathways, either as a pure ultrasound algorithm or in combination with clinical scores or assessments and serves as a guide for further action [39,40]. There are also many indications of the superiority of PoCUS versus the traditional approach and other technologies [19,24,25,31,41,42,43]. Unfortunately, we do not have enough studies that show a benefit of PoCUS and EMUS on the clinical outcome of patients. Nevertheless, there are some reports on their effects on mortality and hospital length of stay or stay in the emergency room [40,44,45,46]

Finally, it should be mentioned that, to our knowledge, no studies have shown a negative effect on the outcome of patients treated with PoCUS.

The major advantages are the speed and mobility not only of the equipment but also the examiners. Furthermore, it can be used in almost all specialties employing diagnostic ultrasound and it can be adapted to individual requirements. It is crucial that internationally recognized standards in education and training programs are available to guide EMUS and PoCUS practice. As already mentioned, many well-established medical societies that are representing specialties with increasing PoCUS or EMUS use have published guidelines and definitions of their ultrasound practice [47,48,49,50,51,52,53,54,55,56,57,58,59]. Other societies, mostly from regions and specialties that do not subscribe to the physician-performed sonography concept, have also attempted to establish definitions of PoCUS and EMUS. These are, of course, valuable attempts by medical societies and specialists practicing within the diagnostic ultrasound realm, but given that there is often extremely limited personal experience of EMUS and PoCUS among these experts, those contributions need to be analyzed with great caution and under the lenses of actual practical experience [49].

In this manuscript, we, as EMUS and PoCUS experts, will focus on definitions of EMUS and PoCUS, as well as current and evolving EMUS concepts.

### 1.2. Current EMUS Concepts

The concept of EMUS is based on the fact that specific clinical questions in emergencies can be answered with the help of sonographic findings and that therapeutic or other consequences can be derived from them. For this purpose, two types of examinations are distinguished: basic, core, or primary; and advanced or secondary exams. The criteria for this distinction are as follows:

− Incomplete to complete coverage of the problem by the sonographic question/s (simple or complex, a single question or multiple yes–no questions, directly or indirectly translatable into the clinical question);
− Degree of difficulty in technical execution;
− Whether rare or common;
− Whether of clinical importance or less relevant;
− Availability of probe and special software (for example, echocardiography).

Because there are different ideas in the emergency community regarding the inclusion and weighting of these five criteria, there is a lack of consensus about what should be considered an indication and assigned to which category (basic or advanced).

Individual EMUS applications are grouped together. The most cited common classification in the literature is one of following five groups by the American College of Emergency Medicine (Table 1) [51]:

1. Resuscitative;
2. Diagnostic;
3. Procedural guidance;
4. Symptom- or sign-based;
5. Therapeutic.

## 2. Evolving EMUS Concepts

Fundamental and critical considerations of the physical examination, especially concerning the use of the stethoscope, a deeper understanding of physical and pathophysiological processes with correlation to sonographic possibilities, technical innovations, and advances in the field of education and training (competence-centered) lead to new concepts and types of applications awaiting their introduction and integration into the practice of emergency medicine.

## 3. Examples of New EMUS Application Possibilities and Concepts

In recent years, many articles have been published on novel applications. They concern nearly all organs and systems of the human body. Only a small part has found its way into routine use. We would like to present two examples: E-FAST, as one of the first indications for EMUS still with potential for development, and pulmonary embolism as a neglected indication. In our opinion, E-FAST needs to be redefined. First, we should go back to the original idea of E-FAST, i.e., to detect major internal bleeding in the trunk and a pneumothorax in unstable patients in less than 2 min. In stable patients, we have more time and can integrate the many technical advances and new indications of ultrasonography into the E-FAST exam. These new applications will overcome the stagnation of E-FAST, which has hampered further development for over 20 years. The pulmonary embolism as a second example shows another problem. The diagnosis of pulmonary embolism (PE) poses a major challenge for emergency departments. The mortality is still high despite the large availability of computed tomographic pulmonary angiography (CTPA), and the risks and problems with CTPA such as radiation exposure, allergic reactions, high costs, and binding of resources, which this technique entails, may not be underestimated. Lung ultrasound, still considered impossible in the 2012 edition of Harrison’s principles of internal medicine, has been used in German-speaking countries for over 30 years for the diagnosis of PE but not in other countries. These two examples are intended to show that PoCUS is still changing and that development has not stood still.

### 3.1. To New Shores in Trauma

**Figure 1:**
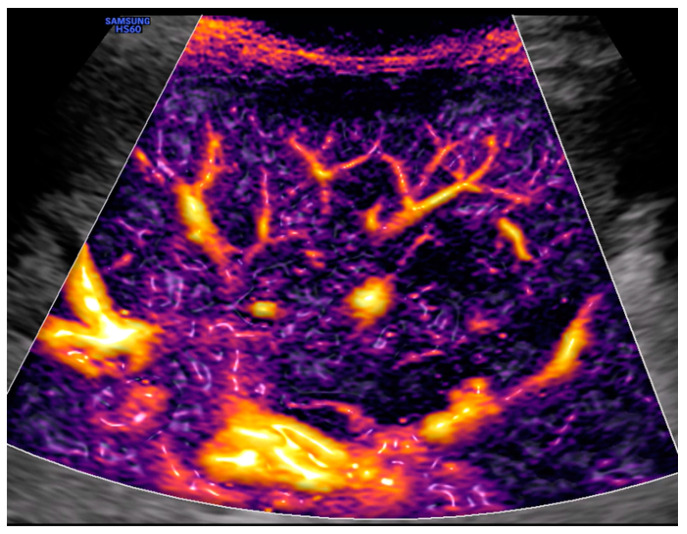
Left upper quadrant view: spleen and Doppler with M-Flow technology. Image courtesy of J. Osterwalder.

**Figure 2:**
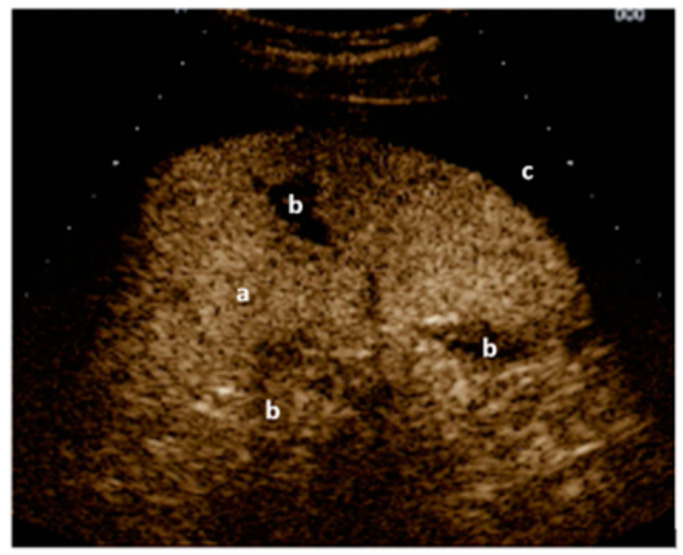
Left upper quadrant view: CEUS of the spleen (a) with black gaps (b) within the spleen (lacerations/ruptures) and around the spleen (free fluid/blood) (c). Image courtesy of J. Osterwalder.

**Figure 3:**
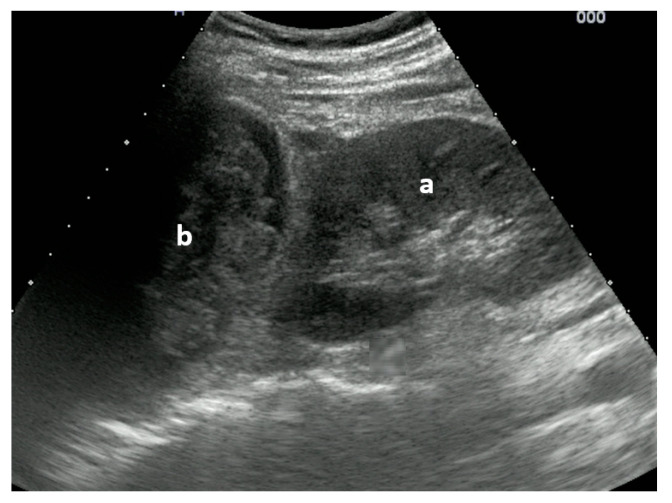
Right upper quadrant view: (a) kidney with traumatic adrenal gland hemorrhage and retroperitoneal hematoma (b). Image courtesy of J. Osterwalder.

**Figure 4:**
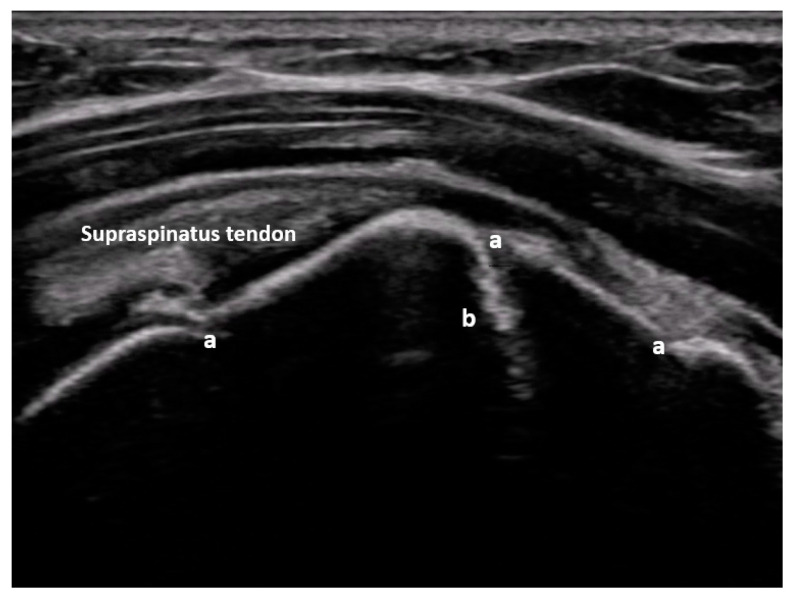
Non-displaced major tuberosity fracture: lateral sagittal view at the level of the major tuberosity with corticalis interruptions (a) and chimney sign (b). Image courtesy of J. Osterwalder.

**Figure 5:**
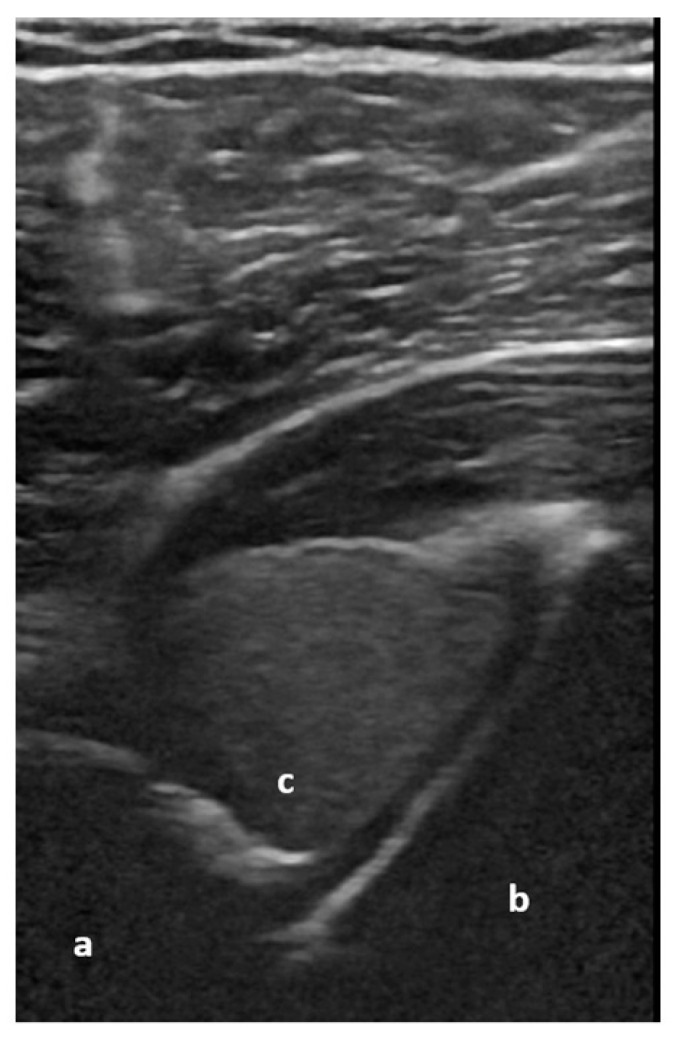
Dorsal transverse view of an anteriorly dislocated humeral head (a), glenoid (b), and hemarthrosis (c). Image courtesy of J. Osterwalder.

The original idea behind the focused assessment with sonography for trauma (FAST) was to quickly detect a possible underlying bleeding into the peritoneal, pleural, or pericardial cavity in unstable trauma patients. With the addition of pneumothorax, the extended FAST (E-FAST) was introduced. Soon, FAST and later E-FAST were expanded to include stable trauma patients and other indications, such as ruptured extrauterine pregnancy or undifferentiated shock [60]. Thanks to improved technology, new modalities, and the growing scientific knowledge of integrating ultrasound into the management of trauma patients, it is now possible to detect even small fluid collections in the peritoneal space, injuries to solid organs with high-sensitivity color Doppler (Figure 1) or contrast-enhanced ultrasound (CEUS) (Figure 2), retroperitoneal hematoma (Figure 3), and free air [61,62]. Many other indications were added over time, such as fractures of the ribs, sternum, and extremity bones (Figure 4), and other musculoskeletal problems and vascular extremity injuries [63,64,65]. Particularly worth mentioning are dislocations of the shoulder (Figure 5), with a rapid reduction in wait time due to not needing to wait for an X-ray to exclude a fracture [37].

There is also a full range of other sonographic examination options available to the emergency physician, which help to work through the primary survey, such as false or main stem endotracheal intubations, pulmonary contusions, hemodynamic evaluation, cerebral hypertension, and cerebral perfusion in traumatic brain injury (TBI) [66,67,68]. Of particular benefit is the hemodynamic monitoring for fluid therapy, the use of vasopressors, and the guidance of invasive procedures [69].

In summary, in addition to the traditional E-FAST examination in unstable patients and an expanded type of E-FAST for stable patients, we see many more fields of application of PoCUS for trauma patients:

1. Expanding E-FAST in stable patients to include additional views for abdominal FAST including the retroperitoneum, evidence of solid organ injury, and free air;
2. PoCUS other than traditional E-FAST such as ABCDE during the primary trauma survey;
3. The monitoring of hemodynamics including cerebral perfusion in TBI to guide fluid and vasopressor treatment;
4. Search for musculoskeletal and soft-tissue or vascular injuries;
5. Guidance with invasive procedures.

#### Pulmonary Embolism

**Figure 6:**
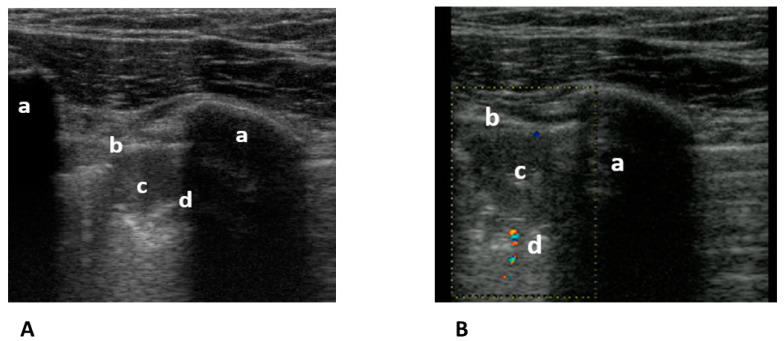
(**A**) Transthoracic longitudinal view: ribs (a), pleural line (b), and subpleural triangular consolidation (c) with central bronchus reflex (d). (**B**) Transthoracic longitudinal view: rib (a) pleural line (b), and second subpleural triangular consolidation (c) with obstructed vessel (d). Image courtesy of J. Osterwalder.

The gold standard for the diagnosis of pulmonary embolism is computed tomography pulmonary angiogram (CTPA). For many years, we have known that lung ultrasound (LUS) can diagnose pulmonary emboli that cause peripheral lung changes. To do so, at least two triangular (Figure 6A,B) or round hypoechogenic subpleural consolidations lacking central perfusion and usually measuring 1–2 cm in size must be detected [70].

The pooled sensitivity of 82% (95% CI 72–88) and specificity of 95% (95% CI 79–95) of LUS for pulmonary embolism in a systematic review and meta-analysis are lower than those of CTPA. The sensitivities vary between 42 and 98% [71]. This wide range is probably the result of different diagnostic criteria for PE, patient selection, and the experience of the examiners. Nevertheless, together with compression ultrasonography of the leg veins and right heart echocardiography (triple sonography), the sensitivity could be increased to 90%, albeit at the cost of a decrease in specificity to 86% [72]. An inhomogeneous meta-analysis on this topic resulted in a sensitivity of 91% and specificity of 81% [73]. However, the reference variable was CTPA in only two studies and clinically derived criteria in five studies. In this context, one paper is interesting. The authors suggested that triple sonography saved 56% of CTPAs because of alternative diagnoses [74]. Despite these encouraging results and the great advantages over CTPAs without radiation exposure, contraindications, the risk of anaphylaxis, high costs, and long waiting times, this type of lung sonography is only offered routinely in a few emergency departments around the world. We believe that it is time to use ultrasound as a diagnostic rule-in tool for PE and thus save some patients and crowded emergency departments from many CTs.

### 3.2. From the Stethoscope to PoCUS

Over 200 years ago, René-Théophile Hyacinthe Laennec invented the stethoscope. Originally, intended only for the heart, it was not until later that the auscultation of the lungs, intestines, and vessels was added. The stethoscope is probably the most widely used diagnostic instrument, but today it is also probably the most poorly used and thus has questionable diagnostic value in terms of its broad impact [75]. Whether the digital stethoscope with a smartphone app will lead to a turnaround in skill and revival is highly questionable. In contrast, PoCUS use, whether in the form of hand-held or small mobile devices, is recommended by many institutions, including the European Federation of Societies for Ultrasound in Medicine and Biology (EFSUMB) [58]. A plethora of studies have shown that US is superior to standard clinical examination and common exam tools, such as the stethoscope and chest and abdominal X-ray, for diagnosing cardiac, pulmonary, and abdominal problems [24,36,41,43,76,77,78].

However, this view is still disputed by some [79]. Contradicting this opinion is the fact that most physicians are poor at using the stethoscope, and the training requires considerable time and effort. Therefore, it makes sense that the paradigm will shift from “stop listening to look” [80]. However, we should not only look at the heart, lungs, abdomen, and vessels, where we have been listening so far, but also exploit the full potential of ultrasound and integrate palpation and percussion, thus redefining the physical examination of the body. Who does not know from experience that when looking for the boundary of lung/diaphragm, apex of the heart, or inferior border of the liver, one is often wrong with percussion/palpation [81]? A myriad of studies exist on this topic, with only one important paper on musculoskeletal indications mentioned here [25]. We believe that PoCUS using a portable or hand-held device could replace significant segments of auscultation exams wherever it is financially viable and should largely replace percussion and palpation. By doing so, we believe that time will be saved, and the use of other unnecessary imaging such as X-ray, CT, or MRI; cost; infrastructure; and human resources can be decreased. While the shift from stethoscope to PoCUS seems more valid than ever, it determining how to integrate it in limited resources regions appears challenging. Although studies are heterogenous and refer to different environments and settings, the development of a tailored curriculum according to the local needs and disease burden, as well as the use of tele-ultrasound training in conjunction with on-site training by POCUS experts are potential solutions in implementing this shift [82]. These are all key concerns in emergency medicine.

This paradigm shift might take place first in well-resourced countries and might begin with student education. The prerequisite is that many medical students already use ultrasound in anatomy, physiology, and pathophysiology courses and are introduced to the basics of its practical application. Clinical examination courses should be supplemented with PoCUS courses for cardiac, pulmonary, abdominal, and vascular examinations. Continuing education in emergency medicine could then focus on specific aspects of emergency medicine. However, physicians in specialty training must also be taught to use handheld or small mobile devices. This not only requires appropriate courses. Even more important is competence-based supervision in everyday practice, which has been largely neglected. This training and its integration into medical education should contain clearly defined learning objectives, accompanied by a structured assessment. The key elements may include the workplace-based supervision of image acquisition and interpretation, formed on relevant clinical indications, integrated in a structured approach within a logical manner and finally incorporated in medical decision making. Additional or alternative means of competence-based supervision can be applied remotely, either with on-site or web-based case simulation. New concepts and ideas are urgently needed to realize this complex and central requirement [83].

In regions with limited resources, in place of traditional imaging modalities which are usually based in a hospital-setting and require trained diagnosticians, PoCUS seems to be a feasible alternative. Instead of expensive medium- and high-end devices, investments should be made in small mobile and handheld devices that cost a fraction of the costs of cart-based systems or existing devices, and most importantly can be used in austere environments by the treating physician [84].

### 3.3. PoCUS-Ultrasound Visualization of Pathophysiological Processes and Correlation of Lung Ultrasound Artifacts with Pathological Lung Changes

Important topics in emergency medicine, such as fluid therapy and pulmonary edema and viral pneumonias, benefit from the correlation of new or deeper knowledge in US physics and pathophysiology. From this, new or improved diagnostic ultrasound concepts are emerging. For example, it has been increasingly recognized that the increase in cardiac output with fluid depends substantially on venous return [85]. Venous return can be easily and adequately described by the Doppler spectrum of large veins, particularly the hepatic vein [86]. Thus, the concepts of volume responsiveness can be combined with volume tolerance and intolerance [87]. In the field of lung sonography, the main concern is the so-called B-lines as vertical US artifacts, which occur at the interface of the pleura and the ventilated lung. Currently, a recent position paper of the World Federation for Ultrasound in Medicine and Biology (WFUMB) makes a clear distinction between artifacts that result from increased water retention in the lung and other parenchymal processes that do not lead to consolidation, such as interstitial lung pathologies [34,88].

### 3.4. Technical Innovations

Elastography: It has matured technically and has been utilized for over 20 years in medical imaging, mostly though in ultrasound-advanced regions of the world. However, it allows for multiple applications in emergency medicine such as trauma, degenerative musculoskeletal diseases, and pulmonary diseases that result in increased lung density without leading to consolidation [89].

Advanced color Doppler technology or other flow technologies: Because of very high sensitivities, it is now possible to visualize even small peripheral blood vessels, with certain devices even in three dimensions. In our opinion, it allows for the detection of hemorrhage, hematomas, and infarcts in solid organs, and in these cases, it can even replace contrast-enhanced ultrasound (CEUS). Among other things, this revolutionizes E-FAST [90,91,92].

Artificial intelligence (AI): An emergency physician needs significant training and supervision to obtain sufficient ultrasound expertise. Here, AI may be able to help via the incorporation of AI-assisted diagnosis into devices. In particular, deep learning technology and convolutional neural networks are promising. Potential applications include lung artifact sonography and quantitative echocardiography for determining ejection fraction (EF), wall motion abnormalities, volume flow, and valve dysfunction [93].

Cloud-based PoCUS: This is a new type of computing platform that has the advantages of low cost, high reusability, high performance, and easy expansion. This technology is already widely used in all fields of medicine for diagnostics and therapy. Thus, a large volume of imaging can be generated using the characteristics of real-time imaging. Using 5G technology, mobile terminal devices such as smartphones or tablets, which are central elements of the EMUS, allow for fast real-time transmissions. Cloud-based PoCUS has huge potential with many versatile possibilities [94].

**Figure 7:**
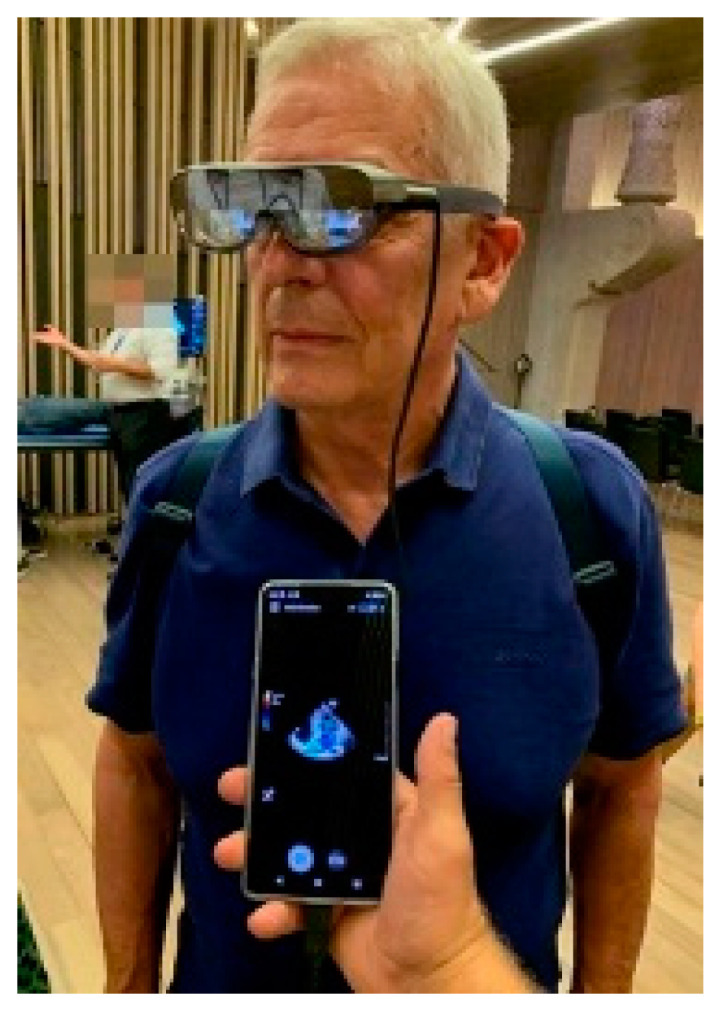
Smart glasses. Image courtesy of J. Osterwalder.

Smart Glasses: Smart glasses are wearable computer glasses that add information to the visual field of the wearer but also can change their optical properties at runtime. These tools are already used in surveillance, security, and navigation, just to name a few areas of application. Augmented reality or virtual reality settings can be achieved. Smart glasses incorporated into PoCUS are already commercially available (Figure 7) and replace hand movements by the operator to adjust machine settings, triggering commands by eye-tracking and eye movements instead of using hands. Other emerging research suggests potential applications in procedural guidance. Here, pilot studies have shown potential promise in ultrasound-guided vascular access via a decrease in the number of cannulation attempts, procedure time, and procedure-related complications rates. These studies used glasses that project real-time ultrasound images over the visual field during the procedure and reduced head movements during interventions [95]. Of course, this technology is emerging and existing literature is still limited.

Future research assessing the additional use of established ultrasound technologies such as elastography or high-sensitive flow will likely emerge when these technologies will be more accessible for point of care settings. AI applications in PoCUS are already on a rapidly increasing trajectory and will likely present one of the fastest growth areas in PoCUS. The speed of advancement in AI technology and effects of AI on medical imaging practice will need to be monitored with the same strict ethical guidance as other AI applications in our daily life, such as driving, design, entertainment, food production, etc.

Future research will improve our understanding of AI’s indications and potential limitations, and how to best implement these exciting new tools.

## 4. Conclusions

Emergency sonography is the application of bedside PoCUS by the attending physician to assist in solutions to as many time-sensitive problems as possible in emergency patient evaluation and management encounters. To date, EMUS encompasses five domains (resuscitative, diagnostic, procedural guidance, symptom- or sign-based, and therapeutic) and two applications are distinguished: basic and advanced. These five domains have been described by many publications and are detailed in several emergency medicine society’s guidelines and position statements [83,96].

Enormous technological advances and scientific findings from the practical application research of PoCUS in traumatic and non-traumatic situations open many new possibilities that are waiting to be introduced and integrated into emergency medicine:

1. There are new indications that concern all organs and systems of the human body. Only a small part has found its way into routine use. The following are two important examples: ultrasound in trauma patients, which should have gone beyond E-FAST long ago, and pulmonary embolism, which is already a standard among experts.
2. As soon as the financial barriers fall, the change from stethoscope, percussion, and mostly palpation, to sonoscope should be implemented as much as possible. That is, the training of clinical examination of medical students must be revolutionized and, wherever possible and appropriate, the already trained physicians must be retrained—a Herculean task.
3. A profound insight into the pathophysiological processes and an understanding of the physical phenomena of ultrasound should be combined. This knowledge must be translated into concepts to better handle hemodynamics, especially volume management, and artifact sonography of the lung (b-lines and elastography to determine lung density).
4. Technical innovations, such as elastography, advanced color Doppler, CEUS and other flow technology, artificial intelligence, cloud-based PoCUS and smart glasses, await their use in emergencies.

## Figures and Tables

**Table 1:** Scope of emergency ultrasound [51].

Resuscitative	Diagnostic	Procedural Guidance	Symptom- or Sign-Based	Therapeutic
Core Applications				
Trauma				
Intrauterine pregnancy				
Abdominal aortic aneurysm				
Cardiac/Hemodynamic assessment				
Biliary tract				
Urinary tract				
Deep vein thrombosis				
Soft-tissue/Musculoskeletal				
Thoracic/Airway				
Ocular				
Bowel				
Procedural guidance				

## Data Availability

Not applicable.
